# Factors associated with the effectiveness of immersive virtual therapy in alleviating depressive symptoms during sub-acute post-stroke rehabilitation: a gender comparison

**DOI:** 10.1186/s13102-023-00742-z

**Published:** 2023-10-20

**Authors:** Karolina Juszko, Pawel Kiper, Adam Wrzeciono, Błażej Cieślik, Robert Gajda, Joanna Szczepańska-Gieracha

**Affiliations:** 1 Faculty of Physiotherapy, Wroclaw University of Health and Sport Sciences, Wroclaw, 51-612 Poland; 2 Healthcare Innovation Technology Lab, IRCCS San Camillo Hospital, Venezia, 30126 Italy; 3 Gajda-Med District Hospital in Pultusk, Pultusk, 06-100 Poland; 4 Department of Kinesiology and Health Prevention, Jan Dlugosz University in Czestochowa, Czestochowa, 42-200 Poland

**Keywords:** Virtual reality, Mental health, Depressive symptoms, Stroke, Neurorehabilitation

## Abstract

**Background:**

The large-scale digitalization of healthcare has induced shifts in patient preferences, prompting the introduction of therapies utilizing novel technologies. In this context, the targeted application of these interventions is deemed as crucial as assessing their overall effectiveness. The aim of this study was to characterize the patient profile who benefited most from immersive virtual reality (VR) therapy.

**Methods:**

Based on the results from the previous randomized controlled trial study, we employed an exploratory study design to determine the factors associated with the most significant mental health improvement. A secondary analysis was conducted on a sample of 83 participants, with further analysis of participants with elevated depression symptoms, as indicated by a score of > 10 on the 30-item Geriatric Depression Scale (GDS-30). Both groups participated in a similar post-stroke rehabilitation program; however, the experimental group also received additional VR therapy through an immersive VR garden intervention. The GDS-30 was used to assess mood and depressive symptoms, and sociodemographic, cognitive status as well as stroke-related variables were considered as potential factors.

**Results:**

In both the experimental (mean change 5.3) and control groups (mean change 2.8), interventions significantly reduced depressive symptoms, with a more pronounced difference in the experimental group (*p* < 0.05). When examining gender differences, women exhibited greater improvement in the GDS, with mean between-group differences of 5.0 for the total sample and 6.0 for those with elevated depressive symptoms. Sociodemographic factors, cognitive status, and time from stroke were not found to be factors that alter the effectiveness of VR therapy.

**Conclusions:**

While VR therapy as an adjunctive treatment for post-stroke rehabilitation seems especially effective for women with elevated depressive symptoms, the results should be interpreted with caution due to the study’s small experimental group size. Traditional methods showed reduced effectiveness in women compared to men; thus, developing technologically advanced and gender-specific approaches can lead to more tailored therapy.

**Trial registration:**

NCT03830372 (February 5, 2019).

## Introduction

Virtual reality (VR) is usually associated with entertainment, but it is also increasingly used in rehabilitation [1]. The rapidly changing world has made life faster and our habits and preferences have changed. Methods that were effective at the end of the 20th century are increasingly less stimulating for today’s patients [2]. Therefore, the medical world began to look for new solutions and new areas of activity, also reaching for VR [3].

Post-stroke rehabilitation should be particularly open to any technical innovations supporting effective recovery, as stroke is one of the main causes of disability [4, 5]. Approximately five million people worldwide suffer permanent disability after stroke each year, with up to 75% requiring assistance from others in their daily activities [6, 7]. The effects of stroke encompass sensory disturbances, motor limitations, and cognitive impairment, along with a diminished capacity for self-care and participation [8]. Additionally, stroke may curtail engagement in leisure activities, leading to decreased psychological well-being, limited social interaction, and an overall reduction in the individual’s quality of life [9]. Post-stroke rehabilitation reaching for VR focuses mainly on therapies that improve motor function. Using interactive video games, the focus is on improving upper limb function, gait speed, balance [10–12].

An important aspect that should not be ignored and can improve the health of stroke patients is their level of psychological well-being. Post-stroke depression (PSD) is recognized as the most common neuropsychiatric complication following stroke [13] and high levels of depression are a predictor of worse rehabilitation effects [14]. However, the use of comprehensive neurological rehabilitation that takes into account the psychological challenges and socioeconomic situation of people after stroke can improve the effectiveness of rehabilitation [15]. VR has already been successfully used in mental disorders [16]. A meta-analysis by Yen and Chiu (2021), also suggests that VR exergames have the potential to positively affect cognitive function, memory and reduce depression among older adults [17]. Other systematic review conducted by Gao et al. (2021), confirms that VR-based interventions used as adjunctive therapy have a positive effect on the treatment of mood disorders and depression in patients with chronic stroke [18]. Training with VR games in stroke patients may also improve psychological characteristics other than depression, such as interpersonal relationships [19].

The sudden emergence of COVID-19 has drawn attention to the problem of patient loneliness and underscored the necessity to develop and implement new digital technologies in the care of acute and chronic patients [20]. It is crucial, in this context, to consider the targeted application of these technologies, as factors such as age, gender, body mass, and cognitive status can influence their effectiveness. For example, among healthy older adults, technological interventions might see a slightly reduced acceptability, attributed to higher dropout rates [21]. In a 2010 meta-analysis, Luppino et al. established a connection between depression and obesity, emphasizing that obesity heightens the risk of depression [22]. Furthermore, approximately 25% of stroke survivors go on to develop dementia, with an even larger percentage experiencing cognitive impairment [23], which can, in turn, influence the risk of post-stroke depressive symptoms [24]. However, within stroke research, gender emerges as a potentially significant factor with a multifaceted impact on depressive symptoms. Eid et al. (2019) concluded in their review that discernible sex differences exist in depression-related gene expression, neuroplasticity, and immune signatures, which may contribute to variations in the prevalence and pathoetiology of the disease between men and women [25]. Additionally, recent knowledge has highlighted the presence of sex-specific variations in the pathophysiology of stroke, emphasizing the importance of integrating gender as a crucial aspect in designing new clinical trials for developing personalized strategies in stroke prevention and treatment [26]. This trend aligns with the growing focus on personalized and precise approaches in post-stroke therapy [27].

Therefore, the present study aimed to is to identify the factors associated with significant mental health improvements following the immersive VR therapy as a method supporting recovery in post-stroke rehabilitation. Accordingly, based on the methodology described and previously used by other authors [28–34], we performed exploratory research including a secondary data analysis of all qualified participants who completed the first phase of the study.

## Materials and methods

### Study design

This study is a secondary analysis of data related to a randomized controlled trial (RCT) evaluating the effects of immersive virtual therapy as a method supporting recovery of depressive symptoms in post-stroke rehabilitation, described elsewhere [35]. While the original RCT explored the effectiveness of VR, this secondary analysis uniquely delves into the factors associated with the effectiveness, which has not been previously analyzed or reported. In the present study, we focus on the first part of the published study design, in which patients underwent a 3-week individual functional rehabilitation (neuro-developmental treatment Bobath concept and proprioceptive neuromuscular facilitation) combined with a VR therapy intervention in the experimental group or Schultz’s Autogenic Training (SAT) in the control group. The entire dataset from the recruiting center for the original research was used in the present study. That study was conducted ethically in accordance with the World Medical Association Declaration of Helsinki; the protocol was approved by the Institutional Review Board at the Wroclaw University of Health and Sport Sciences, Poland (Ref. No. 30/2017) and registered in the ClinicalTrials.gov repository (NCT03830372). All participants were adequately informed about the study and gave their informed written consent to participate.

### Participants

Eighty-three patients with a history of ischemic stroke were initially enrolled in the study and the first analyses were performed on the results of this group. The study excluded individuals with the following conditions and characteristics: epilepsy, vertigo, a Mini-Mental State Examination (MMSE) score less than 24, aphasia, intellectual disabilities, disturbances of consciousness, clinical diagnosis of depression, current use of anti-depressive medication, and undergoing individual psychotherapy. In addition, it was decided to use age above 54 years as an inclusion criterion. The age range was restricted because studies by other researchers confirm that the mental health of stroke survivors differs between young adults and elderly. Young adults (25–54), on average, may have significantly higher depressive symptom scores [36, 37]. The total sample had a mean age of 65.7 years (SD 5.6), an average time since stroke of 4.0 weeks (SD 1.7), and included 48.2% (*n* = 40) women. Table 1 presents the comparative characteristics of both the experimental and control groups, distinguishing between those with and without depressive symptoms.

After initial analyses the original database was revised, and it was decided to use a before the intervention (T_0_) GDS score of > 10 as an inclusion criterion for further analyses. This was dictated by the fact that a score of less than 11 on the GDS suggests the absence of depressive symptoms, so in order to study the effectiveness of therapy, it was decided not to include patients who did not have mental health problems. The final result was a group called the Group with Depressive Symptoms (DS group) and consisted of 60 people, of whom 30 were from the experimental group and 30 from the control group.

**Table 1:** Characteristics of the group, including a comparison of the experimental and control groups

Variable		Total group (*n* = 83)			DS group (*n* = 60)			
		Experimental *(n* = 40)	Control *(n* = 43)	*p*-value		Experimental *(n* = 30)	Control *(n* = 30)	*p*-value
Women, *n* (%)		20 (50.0)	20 (46.5)	0.75^c^		17 (56.7)	13 (43.3)	0.30^c^
Age [years], mean (*SD*)		65.6 (6.6)	65.8 (4.5)	0.82^a^		65.2 (7.3)	65.6 (5.0)	0.82^a^
BMI [kg/m^2^], mean (*SD*)		27.3 (4.9)	27.8 (4.9)	0.48^b^		27.4 (4.6)	28.0 (4.9)	0.61^b^
Education, *n* (%)								
	Primary/vocational	23 (57.5)	28 (65.1)	0.33^c^		17 (56.7)	20 (66.7)	0.36^c^
	Secondary	13 (32.5)	14 (32.6)			9 (30.0)	9 (30.0)	
	Higher	4 (10.0)	1 (2.3)			4 (13.3)	1 (3.3)	
Employment, *n* (%)								
	Disability pensioner	9 (22.5)	4 (9.3)	0.25^c^		6 (20.0)	2 (6.7)	0.30^c^
	Retired	24 (60.0)	30 (69.8)			19 (63.3)	21 (70.0)	
	Employed	7 (17.5)	9 (20.9)			5 (16.7)	7 (23.3)	
Marital status, *n* (%)								
	Single	2 (5.0)	3 (7.0)	0.13^c^		1 (3.3)	2 (6.7)	0.26^c^
	Married	19 (47.5)	27 (62.8)			15 (50.0)	20 (66.7)	
	Widow	19 (47.5)	11 (25.6)			14 (46.7)	8 (26.7)	
	Divorced	0 (0.0)	2 (4.7)			0 (0.0)	0 (0.0)	
Time since stroke [weeks], mean (SD)		3.8 (1.6)	4.3 (1.8)	0.20^b^		3.9 (1.6)	4.0 (1.5)	0.94^b^
Side of the body with paresis, *n* (%)								
	Right	17 (42.5)	15 (34.9)	0.59^c^		12 (40.0)	12 (40.0)	0.79^c^
	Left	23 (57.5)	26 (60.5)			18 (60.0)	16 (53.3)	
	No data	0 (0.0)	2 (4.7)			0 (0.0)	2 (6.7)	
Admitted to the ward from…, *n* (%)								
	Home	36 (90.0)	37 (86.0)	0.99^c^		28 (93.3)	27 (90.0)	0.88^c^
	Hospital	2 (5.0)	2 (4.7)			2 (6.7)	1 (3.3)	
	Other	2 (5.0)	2 (4.7)			0 (0.0)	1 (3.3)	
	No data	0 (0.0)	2 (4.7)			0 (0.0)	1 (3.3)	
Family care, *n* (%)								
	Lack of care capacity	1 (2.5)	2 (4.7)	0.43^c^		0 (0.0)	1 (3.3)	0.52^c^
	Partial lack of care capacity	16 (40.0)	12 (27.9)			11 (36.7)	7 (23.3)	
	Full caring capacity	22 (55.0)	29 (67.4)			18 (60.0)	22 (73.3)	
	No data	1 (2.5)	0 (0.0)			1 (3.3)	0 (0.0)	
MMSE, mean (*SD*)		26.2 (2.5)	27.3 (1.7)	0.07^b^		26.4 (2.3)	27.2 (1.5)	0.22^b^
GDS T_0_, mean (*SD*)		12.1 (4.61)	11.0 (4.6)	0.31^a^		13.8 (4.0)	13.4 (3.1)	0.69^a^

DS group: group with depressive symptoms; *SD*: Standard Deviation, MMSE: Mini-Mental State Examination, BMI: Body Mass Index; GDS T_0_: Geriatric Depression Scale before the intervention; ^a^ according to unpaired *t* test; ^b^ according to Mann–Whitney *U* test; ^c^ according to chi-squared test

### Interventions

Both groups underwent similar upper and lower limb exercises to ensure comparable exercise content, but the training programs were tailored to each patient’s motor capacity with gradual complexity. The treatment focused on restoring functionality of the upper and lower limbs and consisted of 30 min of aerobic training, 30 min of balance exercises, and 60 min of individual rehabilitation following the Bobath concept and proprioceptive neuromuscular facilitation.

**Fig. 1:**
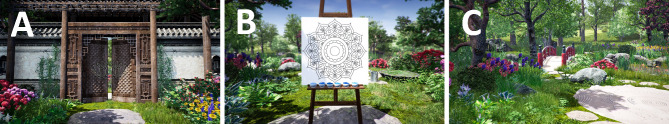
VRTierOne screen captures: **(A)** gate leading to the garden; **(B)** mandala coloring task; **(C)** garden decor elements

The experimental group underwent 10 additional sessions (three times a week, 20 min each) of immersive VR therapy using the VRTierOne device (Stolgraf®, Stanowice, Poland). The hardware used for this therapy included VR HTC VIVE goggles (2017) and two HTC VIVE controllers. The primary aim of VRTierOne was to create an immersive VR experience that would redirect attention to a serene virtual environment, induce a state of relaxation, and aid patients in recognizing their psychological resources. The therapeutic effect of the intervention was based on four key elements, namely, elements of Erickson’s psychotherapy, relaxing music, cognitive stimulation, and a green garden environment (Fig. 1).

Participants assigned to the control group were administered 10 additional sessions (three times a week) of SAT [38]. It is a 20-minute desensitization-relaxation technique, during which participants are instructed to replicate the body relaxation exercises they hear through headphones. This technique follows a universal approach and is suitable for any situation where patient calmness and tranquility are beneficial [38].

### Measurements for the analysis

Outcomes were assessed at two time points: on the patient’s second day on the ward (T_0_, before the intervention) and after three weeks of rehabilitation (T_1_, after the intervention). The difference between the first and second measurement (Δ) was used to determine the effect of the intervention, a positive result meant improvement, a negative result meant deterioration. The primary outcome measure was the 30-item GDS which is a self-rating screening tool to measure depressive symptoms in older adults [39]. The scale contains 30 *‘yes’* or *‘no’* items, and a score between 0 and 10 indicates the absence of depression, while a higher score indicates depression of increasing severity. The GDS provides high reliability (Cronbach’s α = 0.69–0.99) and validity [40, 41].

### Data analyses

All analyses were performed using Statistica v.13.3 PL (TIBCO Software Inc., United States). Continuous variables are presented as means and standard deviations (SD), and the categorical responses are presented as frequencies and percentages. Prior to analysis, the data distribution was tested for normality using the Shapiro–Wilk test. The unpaired *t* test or Mann–Whitney *U* test were respectively used to investigate the differences between continuous (age, body mass index, time since stroke, cognitive status, mental health) and dichotomous variables such as group and gender. In order to correct for multiple comparisons, the Holm method was applied separately for the ‘Total group’ and the ‘DS group’ comparisons. A chi-squared (*χ*^2^) test was used to compare the experimental and control groups in the categorical variables. The relationship between improvements in mental health and continuous variables (including age, body mass index, time since stroke, mental health baseline, and cognitive status) was examined using the Spearman’s correlation coefficient. A one-way analysis of variance (ANOVA) was used to examine the relationship between mental health improvement and categorical variables (education, employment, marital status, family care, location of the patient before admission). The significance level was set at *α* < 0.05.

## Results

The experimental and control groups were comparable in terms of sex distribution, age, body mass index, level of education, employment status, and marital status. There were no significant differences between the groups in the mean time since stroke, side of the body affected by paresis, patient location prior to admission, and capacity for family care. Both groups also showed similar global cognitive status as gauged by the MMSE and baseline mental health status as indicated by the GDS (Table 1).

### Possible factors Associated with Mental Health Improvement

**Fig. 2:**
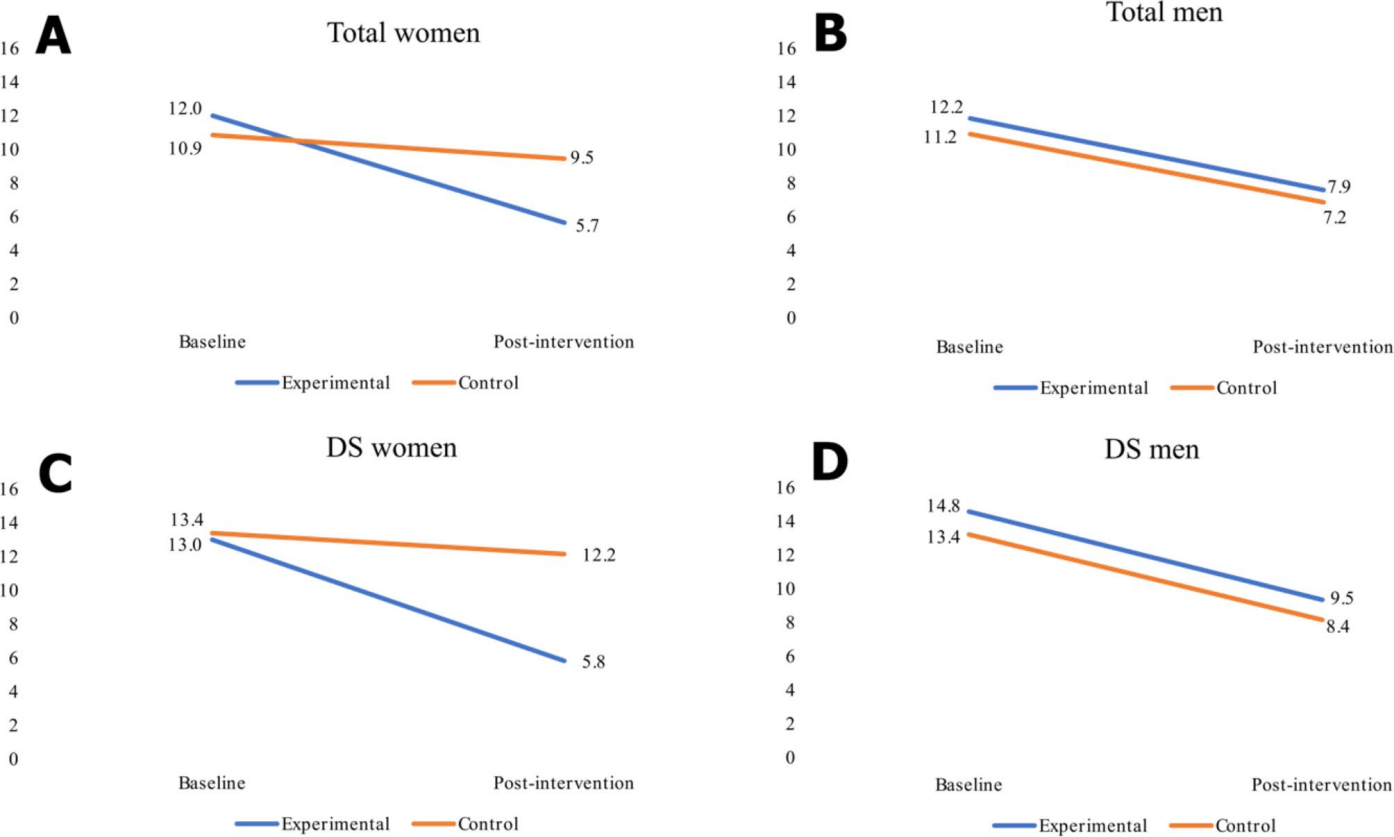
GDS mean values for baseline and post-intervention assessment for **(A)** total women, **(B)** total men, **(C)** women with DS, and **(D)** men with DS.

Using an explorative approach, we employed an unpaired *t*-test to investigate whether gender could be a potential factor associated with improvement. The outcomes in Table 2 confirm the significance of the difference between men and women in control group regarding the intervention’s effect, while Fig. 2 illustrates GDS mean values categorized into four groups: total women, total men, women with depressive symptoms, and men with depressive symptoms. Analyzing the results for the DS group, the mean change in the experimental group was 7.2 (SD 2.5) for women and 5.2 (SD 2.4) for men. In the control group, the mean change was 1.2 (SD 3.8) for women and 5.1 (SD 4.1) for men. The mean improvement in mental health was higher in in men in the control group (*p* < 0.01). While the mean improvement difference between the experimental and control groups was a non-significant 0.1 points for men (p = 0.89), it was a highly significant 6 points for women (p < 0.001).

**Table 2:** Mental Health Improvement in the total studied group and the DS group by gender

		Women		Men		
		*n*	mean (*SD*)	*n*	mean (*SD*)	*p*-value
**Total group**		40	3.9 (4.1)	43	4.1 (3.4)	0.75^a^
	Experimental	20	6.4 (3.3)	20	4.3 (2.4)	0.03^a*^
	Control	20	1.4 (3.2)	23	4.0 (4.0)	0.02^a^
**DS group**		30	4.6 (4.3)	30	5.1 (3.4)	0.60^a^
	Experimental	17	7.2 (2.5)	13	5.2 (2.4)	0.04^a*^
	Control	13	1.2 (3.8)	17	5.1 (4.1)	0.01^a^

DS group: group with depressive symptoms, *SD*: standard deviation; ^a^ according to unpaired *t* test; *non-significant according to the Holm multiple comparison correction

Furthermore, a correlation analysis was conducted to determine potential associations between intervention effects (ΔGDS) and continuous variables. The analysis revealed a significant link between ΔGDS and GDS T_0_ (*r* = 0.57, *p* < 0.05). There were no significant correlations between ΔGDS and other examined parameters, including age, body mass index, time since stroke, MMSE score, education, employment, marital status, family care, or the patient’s location prior to admission.

### Intervention effects

Table 3 presents detailed means and SD for both the experimental and control groups, distinguishing between the total group and the DS group. In total sample, after the intervention, GDS scores decreased by an average of 4 points in the total group, an average of 5.3 points in the experimental group VR therapy and an average of 2.8 points in the control group (standard therapy). The difference between the mean GDS scores after the intervention in the experimental and control groups was not significant (*p* = 0.22). When analyzing the results for the DS group post-intervention (GDS T_1_), mental health status improved on average for both groups, with a statistically significant greater improvement in the experimental group (*p* = 0.03). Furthermore, at T_1_, the average GDS score for the control group was 10, indicating that depressive symptoms remained prevalent on average within this group. The mental health improvement, characterized by the difference (ΔGDS) between the initial (GDS T_0_) and subsequent (GDS T_1_) measurements, was higher in the experimental group by an average of 2.5 points (*p* = 0.002) and 2.9 points (*p* = 0.003) for the total and DS groups, respectively.

**Table 3:** Intervention effects in the total study group and the DS group, including a comparison of the experimental and control groups

		Total	Experimental	Control	Effect size [95% CI]	*p*-value
Total group		*n* = 83	*n* = 40	*n* = 43		
	GDS T_0_	11.5 (4.6)	12.1 (4.6)	11.0 (4.6)	0.22 [-0.21; 0.65]	0.31^a^
	GDS T_1_	7.5 (4.6)	6.8 (3.9)	8.2 (5.1)	-0.16 [-0.39; 0.09]	0.22^b^
	ΔGDS	4.0 (3.7)	5.3 (3.1)	2.8 (3.9)	0.71 [ 0.26; 1.15]	0.002^a^
DS group		*n* = 60	*n* = 30	*n* = 30		
	GDS T_0_	13.6 (3.5)	13.8 (3.9)	13.4 (3.1)	-0.01 [-0.29; 0.28]	0.98^b^
	GDS T_1_	8.7 (4.8)	7.4 (4.2)	10.0 (5.0)	-0.56 [-1.07; -0.04]	0.03^a^
	ΔGDS	4.9 (3.9)	6.3 (2.6)	3.4 (4.4)	0.81 [0.28; 1.34]	0.003^a^

Values are presented as mean (*SD*). DS group: group with depressive symptoms, *SD*: standard deviation, GDS T_0_: Geriatric Depression Scale before the intervention, GDS T_1_: Geriatric Depression Scale after the intervention, ΔGDS: mental health improvement; ^a^ according to unpaired *t* test (effect size given by Cohens); ^b^ according to Mann–Whitney *U* test (effect size given as rank biserial correlation)

## Discussion

The study aimed to identify factors linked to significant mental health improvements following immersive VR therapy in post-stroke rehabilitation. The results indicate that women benefited the most and the only factor associated with mental health improvement of the VR therapy was gender. The female group achieved significantly higher mean improvement after immersive VR therapy than after standard therapy in the control group. In the group with depressive symptoms, women achieved the greatest improvement compared to the control group. This is in contrast to the results from the male group where the mean improvement after rehabilitation was similar in the experimental (VR therapy) and control (standard therapy) groups. These findings are in line with the research on gender role in depression stating that the incidence of depression is closely related to gender, the burden of depression being 50% higher for female than for male [42] with women more likely to experience mood disorders during periods of hormonal fluctuations [43]. Furthermore, all women participating in the analyzed study, in addition to having experienced a stroke, were of postmenopausal age. According to Graziottin and Serafini (2009), postmenopausal depression is more severe than premenopausal depression, has a more insidious course and is more resistant to conventional antidepressants [44].

The causes of the depression’s onset are very complex, and the predicted course of depression is influenced by many factors, including biological, social and psychological factors. In the case of post-stroke depression, the causes are known and according to Erikcens et al. (2016) may be related to the level of physical functioning in the acute phase depending on whether stroke patients live alone and employment status at the time of the stroke [45]. Furthermore, it could be linked to trunk control and the level of basic activities of daily living, particularly in individuals with a higher educational level and cardiac diseases [46]. However, the level of depression is assumed to be stable for the first 18 months after stroke, and fluctuations in post-stroke depression are insignificant during the first two years [45, 47]. At the same time, psychological support that starts in the acute phase and continues throughout the rehabilitation process can be helpful in improving both physical and psychological outcomes after stroke [45]. In our analysis of the experimental group with initial depression symptoms, we obtained an average improvement of approximately 6 points on the GDS. The support provided in this study was relatively quick, with an average of 4 weeks after the stroke incident. This means that, in general, the procedure of immersive VR therapy as a method supporting recovery of depressive symptoms in post-stroke rehabilitation is effective, especially targeted at women. However, more research is necessary to establish the effectiveness level of VR therapy in the men group as in a study of cardiac rehabilitation of male patients, virtual therapy was found to be significantly more effective than traditional methods [48].

The high improvement in mental health in post-stroke patients especially in the female group was related to the original form of therapy using VRTierOne software. The specially created therapeutic game was based on the idea of nurturing a virtual garden and drew on Milton Eriksson’s principles of psychotherapy [35]. The positive role of gardens and green spaces in the prevention of depression has already been demonstrated [49–51] also in earlier studies by the authors [52]. It is noteworthy that the assumptions of Eriksson’s psychotherapy implemented into VR were used for the first time in immersive VR therapy. Thanks to modern technology, the phenomenon of immersion of the senses was achieved by involving not only vision, but also hearing, touch and the vestibular system, all in order to enhance the effect of psychotherapy based on metaphors and symbols. The basic idea of Erickson’s therapy is not to talk about the problem directly but to gain distance from it by using images and words conveying the essence of the problem. One of the tools used in VR therapy is hypnotic suggestions based on the assumptions of positive psychology to help to strengthen the patient’s belief that the healing process has already begun in his or her life and will continue, day by day [53, 54].

The Erickson’s psychotherapy approach differs from cognitive-behavioral therapy (CBT), which is considered a “gold standard” treatment for many individuals. According to Kootker et al. (2017) CBT and computerized cognitive training (CCT) can positively influence the decrease of depressive symptoms in stroke patients [55]. However, secondary analysis of the aforementioned studies showed that one of the variables influencing a lower depression score (as measured by the Hospital Anxiety and Depression Scale) after treatment (CBT group) was male gender [31]. Considering our results, it can be concluded that the type of psychotherapy used influences the effectiveness of treatment by gender. Perhaps the reason for this is that women tend to remember more emotional information than men [56], and therefore, self-help-oriented hypnosis proved to be more effective in the female group.

### Study Limitations

While the results obtained are interesting, this study does have its limitations, and, therefore, they should be interpreted with caution. First, the secondary analysis was based on a small sample size of the experimental group (17 women vs. 13 men). Secondly, only results from one group of neurological diseases were used for the analysis; besides stroke, the most common chronic neurological conditions are Parkinson disease and multiple sclerosis. We suppose it would be scientifically interesting to investigate who benefited most from immersive virtual therapy among such a large group. Thirdly, the study’s analysis relied solely on one research tool (GDS) to assess psychological characteristics. As a result, there is potential value in exploring the impact of immersive VR therapy on other factors such as anxiety, stress, and loneliness, while considering additional covariates like fatigue, motivation, or participation limitations. Finally, the control group in this research received SAT intervention, a relaxation technique without deep therapeutic assumptions. Therefore, future studies should aim to compare the efficacy of immersive VR therapy and traditional psychotherapy based on Erickson’s approach or CBT.

## Conclusions

Immersive VR therapy, when used as an adjunct to post-stroke rehabilitation, appears to offer potential benefits, particularly for women exhibiting more pronounced symptoms of depression. Traditional methods might not yield the same efficacy among female patients as they do with their male counterparts. These variations could be rooted in the distinct ways depression manifests across genders, potentially due to a combination of biological, sociological, and psychological factors. Exploring innovative approaches, while being mindful of these differences, could pave the way for more tailored therapeutic strategies.

## Data Availability

The datasets used and analyzed during the current study are available from the corresponding author on reasonable request.
